# Longitudinal integrated clerkships: a hermeneutic literature review

**DOI:** 10.1007/s10459-026-10500-8

**Published:** 2026-01-16

**Authors:** David L. Garne, Judy R. Mullan, Paul Worley, Ian G. Wilson, Lambert W. T. Schuwirth

**Affiliations:** 1 Graduate School of Medicine, University of Wollongong, Wollongong, Australia; 2 Riverland Academy of Clinical Excellence, Riverland Mallee Coorong Local Health Network, Murray Bridge, Australia; 3 New Medical Education Australia Pty Ltd, Brisbane, Australia

**Keywords:** Longitudinal integrated clerkships, Transformative learning, Relationships, Continuity, Patient-centred care, Student outcomes

## Abstract

The longitudinal integrated clerkship (LIC) approach to medical education, offering students sustained, immersive clinical experiences, is in stark contrast with traditional block rotations. Although evidence shows that LICs are an effective way of educating medical students, much is still unknown on why or how this is so. This hermeneutic review of the literature aimed to produce a synthesis of the pedagogical understanding of LICs by addressing questions about the principles and philosophies of LICs, their intended outcomes, and how student learning is different in LICs compared with more traditional pedagogical structures. This review of 70 peer-reviewed publications identified three core principles underpinning LICs: continuity, meaningful relationships, and immersion in clinical and community settings. These factors may contribute to transformative learning in the form of deeper clinical reasoning, greater patient-centredness, and increased social accountability. Within LICs, the development of long-term, meaningful relationships with the clinical teams in which students are embedded and with patients could be important in fostering the understanding that dealing with patients is an organic, complex process rather than a straightforward mechanistic one. This provides the potential benefit of producing doctors who are more aligned to patient, societal and healthcare needs. The review highlights that LICs could be well-positioned to meet the evolving expectations of modern medical education by producing patient-centred, adaptable, reflective and socially accountable graduates who have the ability to deal flexibly with uncertainty and to be life-long learners.

## Introduction

Longitudinal integrated clerkships were pioneered by the University of Minnesota in 1971 and are thus not new (Zink et al., 2010). However, it took until 2007 for the model to be formally defined by the newly formed Consortium of Longitudinal Integrated Clerkships (CLIC) (Norris et al., 2009). Since then, there has been a rapid growth in the popularity of longitudinal integrated clerkships (LICs), although their implementation varies widely based on a diversity of perspectives and background philosophies. This is not surprising, because such implementations typically need to be highly context-aligned to local societal expectations, local organisational culture, and the available infrastructure and resourcing.

The consensus definition of LICs reached by CLIC in 2007 included that students should:

1. participate in the comprehensive care of patients over time,
2. participate in continuing learning relationships with these patients’ clinicians, and
3. meet the majority of the year’s core clinical competencies across multiple disciplines simultaneously (Norris et al., 2009).

LICs vary in structure, from sequential models with longitudinal themes and limited integration to fully integrated longitudinal models (Hirsh et al., 2007). There is no prescribed duration, with this ranging from as little as eight weeks (Ogrinc et al., 2002) to a median duration of 40 weeks (Norris et al., 2009). Worley et al. (2016) identified three LIC types: A) amalgamative (shorter, partial-discipline models integrated into rotation-based curriculum; B) blended (a hybrid of amalgamated and comprehensive); and C) comprehensive (year long, full-discipline with more limited in-hospital patient exposure). Despite the variations of LIC models, they all share the principles of continuity, relationships and immersion, which are designed to combine to have transformative effects on student learning and thinking.

As medical education is increasingly being required to demonstrate its impact, including on student learning, understanding why this variety exists and the implications for students, clinicians, educators and communities is an important area of enquiry. These questions require engagement not just with *what* LICs achieve, but *why* they achieve what they do, their underlying philosophical assumptions, educations values, and the worldviews that shape their diverse implementations. Different programs appear to emphasise different aspects: some prioritise workforce maldistribution issues, while others focus on transformative learning and explicit beliefs about the value proposition of medical education.

To understand this philosophical variety requires an interpretive approach that allows for engagement with the embedded assumptions within publications. A systematic review may be valuable for aggregating outcome data, but it starts from positivist epistemology focused on standardisation which would require reducing diverse implementations to standardised measures, and through this, losing the contextual richness we sought to understand. A descriptive overview would allow us to report the diversity but not to interpret its meaning or deeper foundations. A critical narrative review could have been considered as it is also aimed at synthesising and critiquing existing evidence and it would be more aligned with an interpretative/(social) constructivist perspective, but its primary purpose is evaluative rather than interpretive.

Therefore, we adopted a hermeneutic approach, a form of narrative review which allows for an interpretive understanding by analysing papers in the context of other papers. This approach takes as its reference point the concept of *verstehen*, the process of creating an interpretive understanding (Greenhalgh et al., 2018). A hermeneutic approach encompasses the following key elements:

- It acknowledges that article texts contain ‘horizons’ or embedded knowledge, assumptions and values, which require interpretation
- It embraces the legitimacy of multiple perspectives rather than seeking a single truth
- It allows iterative engagement with literature as understanding deepens
- It makes more explicit the interpretive role of the researcher in constructing meaning
- It is better suited to understanding why phenomena exist as they do, not just what they are.

Hermeneutics focuses on interpreting texts and embraces continual reinterpretation to develop richer understandings (Illing, 2010). It does not seek a single definitive interpretation but values the evolving nature of understanding. Prentice et al. (2020) describe texts as having ‘horizons’, their embedded knowledge and assumptions, that when engaged with, expand the readers own perspectives. Hermeneutics approaches literature reviews as iterative and dynamic, offering a way of being ‘attuned’ to the literature (Smythe and Spence (2012). Boell and Cecel-Kecmanovic (2014) describe a hermeneutic review as consisting of two interlinked cycles: the first involves searching for and acquiring relevant literature, which is then read, identified and refined; the second is a cycle of analysis and interpretation leading to the development of an argument.

The aim of this hermeneutic literature review was to produce from the published literature a synthesis of the understanding of the underpinning principles and philosophies of LICs, their intended outcomes, and how student learning in LICs is different to that in more traditional forms of medical education.

## Methods

Consistent with hermeneutic review methodology, we began initially by formulating a set of exploratory questions, which were informed by the authors’ experience with teaching and researching in both traditional models and LIC models. These exploratory questions were later aggregated into two key questions:

1. What are the different conceptualisations of LICs, including their underpinning philosophical foundations, principles and intended outcomes?
2. How do students learn during LICs, and how does this differ from learning in the more traditional block rotation (TBR) approach?

### Stages of the review

Aligned with the interactive nature of hermeneutics, our review followed the three stages outlined by Valentine et al. (2021):

1. Literature search and compilation of evidence
2. Data extraction, analysis and interpretation
3. Development of a conceptual model

#### Stage 1: Literature search and compilation of evidence

The search for peer-reviewed, English-language publications on LICs began in 2017. The primary search spanned a 12-month period, with ongoing secondary searches conducted until 2022 as new concepts emerged and new publications appeared. Limiting the search to English was based on the assumption that publications in other languages would be largely based on the English literature because the LIC approach at that time was mainly confined to English-speaking countries (Worley et al., 2016).

We searched the following databases: PubMed, Medline, Web of Science and Academic Search Complete using the terms ‘longitudinal integrated clerkship*’ and ‘longitudinal integrated placement*’. All retrieved papers related to LICs were included without any exclusions. We supplemented this by reviewing reference lists of included papers, and by targeting searches for articles by leading LIC researchers. This led to the inclusion of articles from the psychology and education literature which do not specifically refer to LICs.

One author (DG) conducted the initial search in close consultation with another author (LS). All authors subsequently, at regular monthly meetings, discussed the publications found in the primary and secondary searches. Decisions regarding the inclusion or exclusion of articles were reached by consensus. Articles were excluded if they did not contribute meaningfully to the questions that we were asking. Each included paper was read and analysed in relation to the key research questions listed above. References were collated by publication year and categorised into MS Word_®_ tables, corresponding to each key question.

#### Stage 2: Data extraction, analysis and interpretation

Throughout the review, key questions, findings and arguments were synthesised into the MS Word® tables. The process was iterative, with a constant interplay between search, acquisition, and interpretation cycles. The review concluded upon reaching consensus that saturation had been achieved and at the conclusion of this process our initial questions remained valid. Questions about the how LICs contribute to the development of clinical reasoning were raised by the review process but were considered to be outside the scope of this review.

#### Stage 3: Development of a conceptual framework

A conceptual framework, a key element of hermeneutic reviews, is defined as a network of related concepts that helps us to understand a particular phenomenon (Jabareen, 2009). During the review process, we developed a conceptual framework based on logical inferences derived from the literature and informed by our understanding of the LIC literature. The initial concepts and themes were sourced from the literature to provide input to our two key questions. This provided the basis for the conceptual framework which was developed and will be presented in the results section below.

### Philosophical stance and assumptions

To add transparency to our methodology we make our ontological and epistemological assumptions explicit.

Ontologically we have adopted a constructivist-realist position. We accept that LIC implementations per se exist as real educational structures with measurable outcomes, but simultaneously we recognise that *how* these programs are conceptualised, valued, and their specific implementation choices are socially constructed through the values and meanings attributed by educators, students, clinicians, and institutions.

Epistemologically we have used an interpretivist stance. We recognise that knowledge about LICs is not simply discovered but is constructed through interpretation. We acknowledge that the backgrounds of all authors as medical educators and researchers with experience in both traditional and longitudinal models has shaped our interpretation, and we have deliberately sought to remain open to multiple perspectives and underlying philosophies represented in the literature. The hermeneutic circle, with its iterative movement between parts and whole, guided our analysis and allowed our understanding to evolve as we engaged more deeply with the literature.

We received no funding for this research and have no conflict of interest to declare. DG wrote the initial manuscript draft, and JM, PW and L.S made considerable contributions in revising, condensing and refining the manuscript. All authors reviewed and approved the final manuscript. As it was a literature review, ethics approval was not applicable.

## Results

Of the 212 papers reviewed, 70 were found to contribute to answering the research questions and were included in this review. See Table 1 for a summary of the included publications.

**Table 1:** Summary of papers included in the literature review

**Underpinning philosophical views of LICs**	
Deontological view	Worley et al. (2000); Hirsh et al. (2007); Walters et al. (2012); Teherani et al. (2013); Girotti et al. (2015).
Teleological view	Eva (2005); Walters et al. (2012); Kelly et al. (2014).
**LIC principles**	
Continuity	Hirsh et al. (2007); Wamsley et al. (2009); Couper et al. (2011); Stevens et al. (2014); Ellaway et al. (2016); Asgarova et al. (2017); Weston et al. (1989); Walters et al. (2012); Campbell et al. (2017); Irby (2007); Hauer et al. (2014); Hauer et al. (2012); Hirsh et al. (2012); Worley et al. (2006); Stolper et al. (2021); Kulasegaram et al. (2017); Hatala et al. (2003); Rohrer (2012); Worley et al. (2000); Bartlett et al. (2017); Lave and Wenger (1991); Hauer et al. (2014); Hauer et al. (2012); Hauer et al. (2012); Bonney et al. (2014).
Relationships	Worley et al. (2006); Stolper et al. (2021); Christakis and Feudtner (1997); Campbell et al. (2017); Fuller, Lawson & Beattie (2021); Hirsh et al. (2007); O’Doherty et al. (2022); Daly et al. (2013); Wenger (2000); Roberts et al. (2017);
Immersion	Worley et al. (2000); Bartlett et al. (2017); Lave and Wenger (1991); Hauer et al. (2014); Hauer et al. (2012); Hauer et al. (2012); Bonney et al. (2014).
**Student learning during LICs**	
Multiple repeated exposure to clinical cases	Zink et al. (2008); Shahi et al. (2015); Roberts et al. (2017); Campbell et al. (2017); Latessa et al. (2017); Worley et al. (2004b); Rohrer (2015); Chamberland et al. (2015); Hemmer et al. (2015); Schmidt and Rikers (2007); Ericsson (2007).
Encoding specificity	Salzberg (1976); Epstein et al. (1979); Staudigl and Hanslmayr (2019); Regehr and Norman (1996).
Retrieval of information from long-term memory	Postman and Warren (1972); Riley and Riley (1996); Regehr and Norman (1996); Postman and Weingartner (1969).
Mixed or interleaved learning	Kulasegaram et al. (2017); Rohrer (2012); Hatala et al. (2003); Rohrer and Pashler (2010); Hruska et al. (2016); Taylor and Rohrer (2010); Worley et al. (2004a); Ogur et al. (2007); Hansen and Simanton (2009); Poncelet et al. (2011).
**Intended outcome of LICs**	
Medical education imperatives	Ogrinc et al. (2002); Whitcomb (2005); McLaughlin et al. (2011); Huang and Malinow (2010); Konkin and Suddards (2012); Gaufberg et al. (2014); Walters and Brooks (2016).
Workforce imperatives	Roberts et al. (2012); Playford, Ng, and Burkitt (2012); Weston et al. (2018); Amin et al. (2018); Eley et al. (2012); Figueiredo et al. (2019); O’Sullivan and Chater (2019).
Patient and population imperatives	Medalie et al. (1997); Bell et al. (2008); Teherani et al. (2009); Daly et al. (2013).

**Fig. 1:**
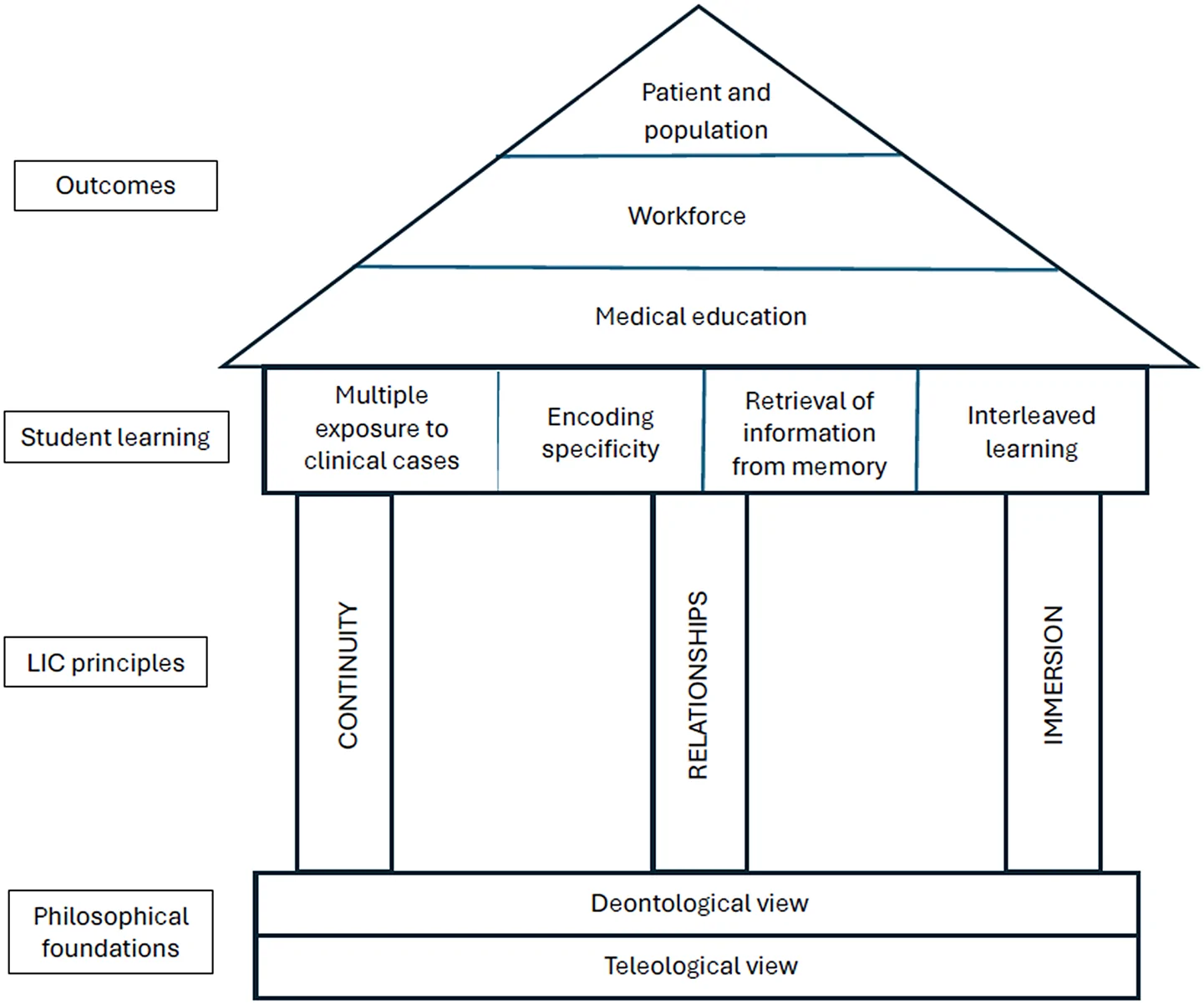
Conceptual framework of LICs

The conceptual framework that emerged from the interpretive process of this hermeneutic review reflects our synthesis of the diverse philosophical horizons encountered across the literature, and a graphical representation of this framework is presented in Fig. 1. It shows the philosophical foundations upon which the overlapping LIC principles of continuity, relationships and immersion are built, and how this influences student learning. This learning then leads to the pinnacle of the intended outcomes of LICs. The results are presented in four sections which align with our conceptual framework.

### Underpinning philosophical foundations

The various models of LICs share two overarching philosophical views, a deontological view and a teleological one, which support their underlying principles. These two views are not mutually exclusive and it is possible, and indeed desirable, for elements of both to co-exist simultaneously and to be complementary.

The deontological, or value-based view, emphasises exposing students to a patient-centred approach and social accountability as moral imperatives, shaping their attitudes to patients and careers (Worley et al., 2000; Hirsh et al., 2007; Walters et al., 2012; Teherani et al., 2013; Girotti et al., 2015). This view sees exposing students to primary care, and especially rural primary care, is important as a deontological moral and educational imperative.

The teleological, or pragmatic view, focuses on the cognitive adaptability in thinking and reasoning fostered by LIC models, allowing students to apply more bespoke clinical solutions to their patients, thereby potentially improving clinical reasoning (Eva, 2005; Walters et al., 2012; Kelly et al., 2014). This also relates to exposing students to primary care, especially rural primary care, with the teleological aim to better equip them for medical practice in these settings.

### LIC principles

Continuity, a fundamental principle of LICs, encompasses continuity of supervision, patient care and curriculum, and supports the development of student idealism (Hirsh et al., 2007; Wamsley et al., 2009; Couper et al., 2011; Stevens et al., 2014; Ellaway et al., 2016; Asgarova et al., 2017). It has the potential for students to engage with comprehensive patient care over time, shifting the learning focus from disease to illness, and this may support a deeper understanding of chronic illness management (Weston et al., 1989). Focusing on symptomatic disease, commonly taught in TBRs, may ignore the equally important realities of asymptomatic disease (e.g., undiagnosed hypertension) and illness without disease (e.g., grief). By contrast, this continuity triad found in LICs can shift the learning focus from the disease to the patient. Continuity can promote stronger connections between students and patients, between students and supervisors, and between the medical sciences and clinical medicine, and this, in turn, can foster greater patient-centredness and learner-centredness (Hirsh et al., 2007). The sharing of patients over time by supervisors and students may foster patient-centred learning and the sense of being known by the supervisor over time can foster learner-centred teaching.

Hirsh et al. (2007) and Walters et al. (2012) argue that learning should be embedded in patient care rather than disease, enabling students to understand illness within the broader context of patients’ lives. LICs foster this approach by promoting continuity of supervision and of patient care, which has the added potential benefit of supporting constructive global feedback and early identification of struggling students (Campbell et al., 2017). By way of contrast, TBRs are characterised by what Irby (2007) describes as discontinuity. These short-term, often impersonal relationships with supervisors, patients, and place can lead to anxiety and frustration among students which can have negative impacts on their learning (Hauer et al., 2014, 2012a; Hirsh et al., 2012; Irby, 2007).

LICs may support students in developing deeper, more meaningful relationships with their supervisors, patients and communities – factors shown to have a beneficial effect on student engagement and learning (Worley et al., 2006). This is especially applicable in primary care settings, where students are more likely to be exposed to patients’ lived experiences and integrate these into clinical reasoning processes (Stolper et al., 2021). With increasing medical specialisation and shorter hospital stays for complex medical problems, trainees in these settings often do not see in the supervisors or get the opportunity to practice themselves the personal touch that is so often needed by patients. Christakis and Feudtner (1997) noted that the ‘healing touch’ in major medical centres is fleeting, and trainees’ have more limited ability to build long-term relationships with patients and their treating teams. This could lead to functional, short-term encounters rather than more meaningful relationships, a concern expressed nearly three decades ago that likely persists today.

LICs tend to integrate students into clinical teams and expose them to longer-term doctor-patient relationships, especially in rural settings, which may have beneficial outcomes with regards their learning. This is a major shift from the situation encountered by students in major teaching hospitals. Campbell et al. (2017) found that the continuity of supervision which LICs provide can allow students who are not progressing as expected to be identified early. It provides capacity for students to develop deeper, more meaningful relationships with their clinical supervisors which may also have a beneficial effect on student learning (Worley et al., 2006). Fuller, Lawson & Beattie (2021) found that these stronger relationships can lead to greater entrustment by supervisors and deeper student involvement in patient care.

The continuity of patient care, curriculum and clinical supervision may support student learning during LICs (Hirsh et al., 2007). Strong relationships within LICs can foster social learning systems, enabling students to engage in multiple communities of practice and develop clinical skills and professional identity (O’Doherty et al., 2022; Daly et al., 2013). LICs may lend themselves more readily to students becoming legitimate peripheral participants in teams caring for patients, a theory proposed 25 years ago (Wenger, 2000; Lave & Wenger, 1991; Hauer et al., 2014). Connectivity is seen as an important enabler of this process (Roberts et al., 2017), which is more limited in TBRs. LIC students, once initially settled into a single program, may benefit from less interruption in their learning. Conversely, students in short, disconnected rotations may repeatedly face high cognitive loads during the initial stages of each rotation, potentially disrupting their learning and development.

Beyond continuity and relationships, LICs offer deep clinical immersion, much deeper than is possible in short-term rotations (Worley et al., 2000; Bartlett et al., 2017). Students are integrated into clinical teams, gaining acceptance as legitimate contributors, and this immersion enhances placement satisfaction and learning outcomes (Hauer et al., 2012, 2012b). Longer clerkships can also foster meaningful community engagement, which can allow students to build local connections not possible in shorter placements (Worley et al., 2000; Bartlett et al., 2017). Bonney et al. (2014) found that students in one LIC program who engaged more deeply with their communities not only reported greater satisfaction but also reported enhanced learning and a strong sense of belonging.

### Student learning during LICs

Compared to TBR programs, LICs can enhance student learning through four overlapping domains: (1) multiple repeated exposure to clinical cases; (2) encoding specificity; (3) retrieval of information from long-term memory; and (4) mixed or interleaved learning.

#### Multiple repeated exposure to clinical cases

Medical students in LIC programs can engage with more patients, especially undifferentiated patients, than students in TBR programs (Zink et al., 2008; Shahi et al., 2015; Roberts et al., 2017; Campbell et al., 2017; Latessa et al., 2017; Worley et al., 2004). While students in TBR programs often see patients after diagnosis and have more limited interactions with them, LIC students can encounter complex, evolving cases and often follow patients over time, gaining insight into disease progression and resolution. It also may allow students to see repeated similar patient presentations which has been shown by psychological research to reinforce concepts in long-term memory – so-called spaced learning (Rohrer, 2015).

LICs can allow students to have authentic roles within clinical teams (Roberts et al., 2017), fostering clinical reasoning through repeated exposure to clinical cases, problem solving and reflection (Chamberland et al., 2015). Frequent patient contact may further enhance academic achievement (Hemmer et al., 2015), and may support the development of encapsulated knowledge and illness scripts (Schmidt & Rikers, 2007).

Immediate feedback is essential for expertise development (Ericsson, 2007). This is a key feature of LICs, supported by strong student-preceptor relationships. LICs are often based in community primary care settings, providing scope for seeing patients with broad diagnostic possibilities, thus enhancing clinical reasoning (Campbell et al., 2017). The parallel consulting model, combined with repeated exposure to cases, can promote iterative and non-linear thinking. LICs, therefore, may mirror the complex, evolving nature of clinical reasoning more closely than TBRs, which tend to be more brief, linear and mechanistic.

#### Encoding specificity

Encoding specificity refers to improved memory retrieval when learning and application contexts align (Salzberg, 1976; Epstein et al., 1979; Staudigl & Hanslmayr, 2019). Learning clinical context directly from the patient encounter enhances recall more effectively than textbook-based learning. This supports the value of community-based LICs, where students can learn in real-world settings that mirror future practice (Regehr & Norman, 1996). By way of contrast, traditional hospital-based education often occurs in environments where patients are isolated from their rich, authentic contexts, thus limiting encoding specificity.

#### Retrieval of information from long-term memory

The total time hypothesis suggests learning is directly and linearly related to the total amount of time spent learning (Postman & Warren, 1972; Riley & Riley, 1996), and frequent retrieval improves long-term memory (Regehr & Norman, 1996). LICs with their integrated and ongoing exposure across disciplines, can support both. The vaccination theory of Postman and Weingartner (1969) warns against static learning that once tested, students may disengage. LICs counter this by requiring repeated, unpredictable use of knowledge, acting as cognitive ‘booster shots’ that reinforce learning.

#### Mixed or interleaved learning

Mixed or interleaved learning involves students learning and practicing multiple concepts together or cumulatively, rather than in isolation. Research shows that this approach facilitates the transfer of learning (Kulasegaram et al., 2017; Rohrer, 2012; Hatala et al., 2003). Learning is most durable when study is distributed over time, and interleaving different types of practice problems markedly improves retention (Rohrer & Pashler, 2010). Hruska et al. (2016) hold the view that dispersed learning and repeated testing may refine knowledge structures and improve long term memory.

Experimental psychological research shows that interleaved learning can improve test scores when compared with block learning (Taylor & Rohrer, 2010). In medical education, students in LIC models, where learning is interleaved, often perform better in examinations than their TBR counterparts (Worley et al., 2004; Ogur et al., 2007; Hansen & Simanton, 2009; Poncelet et al., 2011).

LICs can expose students to concepts in random, unpredictable and repeated ways, unlike structured, time-limited, specialty TBR rotations. This, then, leaves little doubt that specialty TBR rotations offer much more limited opportunities for interleaved learning across multiple concepts.

In summary, all four of the categories listed above are characteristics commonly found in LICs but are much less common in the TBRs which take place in large academic medical centres. These four categories describe the major differences in learning by students in LIC and TBR models.

### Intended outcomes of LICs

While LICs were initially developed to address rural workforce shortages, their impact may extend to broader domains including medical education, and patient and population imperatives (Zink et al., 2010; Halaas, 2005; Halaas et al., 2008). As medical care has moved progressively from hospital to community settings, the training of medical students has followed (Ogrinc et al., 2002). Regardless of the medical education program models, the goal remains to prepare work-ready interns with broad clinical competence. However, increasing sub-specialisation and shorter hospital stays have limited student exposure to diverse clinical experiences in hospital settings, potentially impacting on training quality (Whitcomb, 2005). Although in writing this Whitcomb was focusing on the North American model, it would likely apply anywhere in the developed world. LICs counter this by replacing specialist-heavy hospital rotations with generalist-led and often community-based training. This model can offer broader clinical exposure and may provide graduates with the broader base of skills required for work as junior doctors

(McLaughlin et al., 2011).

LICs can influence the type of professional identity that students develop during these programs. Emerging evidence shows that students in LIC programs can become more reflective, patient-centred, socially accountable and engaged in patient advocacy (Huang & Malinow, 2010; Konkin & Suddards, 2012; Gaufberg et al., 2014; Walters & Brooks, 2016). Gaufberg et al. (2014) found that these traits not only developed during LICs but persisted for least 4–6 years post-completion.

Evidence suggests that LIC exposure, especially in rural settings, can encourage students to pursue careers in areas of workforce need (Roberts et al., 2012; Playford et al., 2016; Weston et al., 2018). LICs may also promote interest in primary care through sustained engagement during training (Amin et al., 2018). Both LICs and traditional rural placements can expand students’ awareness of career options (Eley et al., 2012), often fostering interest in rural career options (Roberts et al., 2012). Additionally, training in underserved regions has the capacity to inspire local youth by showcasing local educational opportunities (Figueiredo et al., 2019; O’Sullivan & Chater, 2019) TBR-based programs in hospital departments and/or clinical specialties often prioritise departmental or specialty needs rather than that of students or patients (Medalie et al., 1997). Students can experience brief, fragmented interactions with medical teams and patients (Bell et al., 2008), potentially limiting their preparedness for managing complex healthcare needs in ageing populations (Teherani et al., 2009). By way of contrast, LICs can offer sustained hands-on learning that fosters understanding of patients’ cultural and contextual backgrounds, enhancing health care quality. This model can promote community service, enhances student self-efficacy, and aligns graduates with societal, healthcare system and individual patient needs (Daly et al., 2013).

## Discussion

Although LICs have been utilised in medical education for more than 50 years, it is relatively recently that their popularity has increased, and consensus has been reached about how they are defined. Despite this agreement, implementation of LIC programs varies considerably, based on local expectations, culture, infrastructure and resources. In this review, we sought to move beyond simply describing what LICs achieve to interrogating why and how they work. Because of the variation in conceptualisations and implementations of LIC programs, we considered that a hermeneutic approach would provide the richness of what we were seeking to understand. The conceptual framework that developed from our questions and from reviewing the literature provided a solid structure to further interrogate the literature and to frame our findings.

The deontological (value-based) and teleological (pragmatic) views, although not unique to LICs, are strongly represented, and we argue that this may be why students in LICs appear to develop a more patient-centred approach to their practice and have a stronger sense of social accountability.

There is an implicit understanding in the LIC context that dealing with patients is an organic, complex process rather than a straightforward mechanistic one. Many LICs happen in a patient-centred context with continuity of place and curriculum, together with the potential ability to develop long-term, meaningful relationships with clinical supervisors and patients. We argue that this may provide the added benefit of producing doctors who are more aligned to patient, societal and healthcare needs. However, because LIC models of medical education are relatively new, less research has been conducted on students engaged in these programs and much is still unknown about how and why they impact healthcare needs.

The pillars of continuity, relationships and immersion found in LICs, which set them apart from more traditional medical educational models, appear to promote a different approach to learning. These include the capacity of students to see more clinical cases (especially undifferentiated ones), the interleaved manner of learning that occurs (with its associated variability and unaggregated learning), the encoding specificity that can develop, and the retrieval of information from long-term memory.

According to Greenhill and Poncelet (2013), the LIC model is underpinned by two theoretical concepts: continuity and symbiosis, both of which serve to have a transformative effect on the thinking and attitude of student participants. This has a foundation in Mezirow’s transformative learning theory (Kitchenham, 2008). Continuity of place can have a mitigating effect on the repeated disorientation with which students are often faced with each change of rotation, which can be overwhelming from a cognitive load perspective. In the LIC model, students can become legitimate peripheral participants in the treating teams in which they are placed rather than being external bystanders, and they thus feel more supported and their learning is more active and agentic.

A further theoretical concept relevant to the learning that can occur during LICs is that of social learning systems or the legitimate peripheral participation theory originally developed by Wenger. He framed learning as a social process which is the interplay between social competence and personal experience (Wenger, 2000). This involves students becoming legitimate peripheral participants in the delivery of healthcare, participating in multiple communities of practice, negotiating the boundaries between these, and using this to develop their own individual personal and social identities (Daly et al., 2013b).

LICs aim to transform student perspectives. Evidence shows that LIC students may maintain stronger patient-centred attitudes (Teherani et al., 2013), and they are more likely to take on advanced patient-care roles (Hauer et al., 2012b). Walters et al. (2012) found that that LICs may enhance clinical, teamwork and patient-centred skills. LICs can also transform how students view patients as people and members of families and communities rather than simply as a disease. This shift may foster stronger social accountability, influencing future career paths (Walters et al., 2012; Hirsh et al., 2012; Greenhill & Poncelet, 2013).

It could be argued that TBR structures allow students to adapt better and more flexibly to new, unfamiliar work environments. This review, however, did not seek to address the issue of adaptation, specifically not if it comes at the expense of more active learner engagement. It could also be argued that LIC learning environments have the potential to create difficulties for students who are not highly motivated and not self-directed learners, as well as the assessment bias that is possible when students are assessed by preceptors with whom they have strong relationships. Once again, this review did not attempt to address these issues – these could be fruitful areas for further research. A question that arises from this review relates to the capacity of LIC students and graduates to develop good clinical reasoning. This, too, was outside the scope of this review, but is also a topic that is ripe for exploration in the future.

A strength of this review is its breadth, rather than restricting the search to a particular time period. We reviewed the literature as far back as the searches led – in this case more than 50 years. This comprehensive approach, combined with snowballing techniques, uncovered relevant articles not only within medical education but also across psychology and education fields. Another strength lies in the expertise of the authors, all of whom have been directly involved in both traditional and LIC forms of medical education, which has provided firsthand insights into both forms of education. At the same time, all authors were conscious that this could lead to bias, and addressed this though ongoing reflexivity, with regular discussions and constant scrutiny throughout the review process.

One limitation of this review is that we limited our search to articles written in the English language only, which may have resulted in the omission of some relevant publications. However, given that LIC programs were at the time of the primary search, mainly confined to English-speaking countries, any publications in other languages would be largely based on the English literature. A further potential limitation was our decision to develop our own conceptual framework rather than adopting an existing theoretical model. We have however, described this conceptual framework development process clearly, and invite the reader to judge the validity and utility of our approach.

## Conclusions

It is becoming apparent that more traditional forms of medical education may no longer be fully aligned to societal expectations of the modern doctor. Contemporary medical education needs to produce graduates with the ability to be patient- and community-centred, to deal more flexibly with uncertainty, and to be life-long learners. From this study, we argue that LICs may be better placed than TBRs to achieve this. This review suggests that the combination of principles, structures, experiences and outcomes that LICs provide has the potential to have important and transformative effects on the thinking, learning and decision-making of students and graduates.

## Data Availability

No datasets were generated or analysed during the current study.
